# Type I IFN receptor blockade alleviates liver fibrosis through macrophage-derived STAT3 signaling

**DOI:** 10.3389/fimmu.2025.1528382

**Published:** 2025-04-07

**Authors:** Soo-Jeung Park, Josefina Garcia Diaz, Tina Comlekoglu, Young S. Hahn

**Affiliations:** ^1^ Beirne B. Carter Center for Immunology Research, University of Virginia, Charlottesville, VA, United States; ^2^ Department of Microbiology, Immunology and Cancer Biology, University of Virginia, Charlottesville, VA, United States

**Keywords:** liver fibrosis, IFNAR-1, Kupffer Cells, stat3, tissue repair

## Abstract

Liver macrophages play a role in the development of liver fibrosis progression via the regulation of inflammatory signaling. However, the precise mechanisms of macrophages contributing to liver fibrosis progression remain unclear. Using a preclinical model of CCl4-treated mice, we determined the composition of immune cells and the alteration of inflammatory gene expression. Our findings revealed a significant increase in liver macrophages, particularly those derived from infiltrating blood monocytes, in fibrotic mice. Moreover, the expression levels of type I IFN signature genes such as *IFNα*, *IFNβ*, *ISG15*, *USP18*, *Ifi44*, *Ifit1*, *Ifit2*, *IRF3*, and *IRF7* were elevated in fibrotic mice. To determine the role of type I IFN signaling in liver fibrosis, we administered an IFNAR-1 antibody to block this pathway for 3 days prior to harvesting the liver. Notably, IFNAR-1 blockade reduced macrophage numbers compared to control mice and alleviated liver fibrosis in mice with increased hepatocyte proliferation and apoptosis. The ratio of P-STAT3/P-STAT1 in monocyte-derived macrophages was increased in the IFNAR-1 blockade group compared to fibrotic mice, and this was related to the appearance of M2 macrophage differentiation. Additionally, single-cell RNA-seq analysis indicated that IFNAR blockade affected inflammatory pathways involved in hepatocyte regeneration and fibrosis prevention. Taken together, IFNAR-1 blockade alleviates liver fibrosis progression by modulating macrophage inflammatory responses. These results provide insights for developing anti-fibrotic therapies against type I IFN signaling.

## 1 Introduction

Liver fibrosis is a global health problem and demands medical attention (1). In the United States, the treatment of advanced liver fibrosis incurs significant costs, contributing to an economic burden of $96.18 billion (2). Liver fibrosis arises from excessive scar tissue formation in the liver due to chronic injury or inflammation. It is often caused by hepatic viral infection, alcohol, and non-alcoholic fatty liver disease (NAFLD). The prevalence of NAFLD among U.S. adults is projected to rise from 27.8% in 2020 to 34.3% by the year 2050, exacerbating the fibrosis problem. If liver fibrosis is not controlled, it can progress to severe liver diseases such as cirrhosis and hepatocellular carcinoma (HCC) (3). During the repair process, the liver accumulates extracellular matrix (ECM) proteins, leading to an uneven surface texture and fibrotic scar formation (4). Hepatocyte proliferation and recovery are mediated by interactions with immune cells, particularly macrophages, which are known to undergo metabolic reprogramming (5). While numerous animal models of liver fibrosis have been developed for studying liver fibrosis, carbon tetrachloride (CCl_4_) remains a well-established model for elucidating liver damage mechanisms (6).

Type I interferons (IFNα and IFNβ) play a role in antiviral responses and immune regulation. They bind to the type-I IFN receptor (IFNAR), composed of IFNAR1 and IFNAR2, to induce the transcription of interferon-stimulated genes and influence T cell responses and dendritic cell maturation (7). The signal transducer and activator of transcription1 (STAT1) can be activated by Janus kinase (JAK) in canonical IFN signaling and induce proinflammatory factors (8). STAT3 is a transcription factor that regulates cell growth and cell survival, while modulating the switch from pro- to anti-inflammatory signaling (9). Upon binding of a cytokine like IL-6 to IFNAR, JAK activation leads to STAT3 phosphorylation, forming homodimers or heterodimers that translocate to the nucleus. These dimers bind to STAT-binding elements (SBEs) in the promoters of target genes (10, 11). Type I IFN can exacerbate liver damage by enhancing the TLR4 response in liver macrophages and promoting pathogenic CD8 T cell accumulation in fatty liver disease (12, 13). Increased expression of type-I IFN has been noted in immune cells within the portal tract of primary biliary cholangitis, an immune-mediated cholestatic disease (14). Increased type I IFN expression has been observed in immune cells within the portal tract of primary biliary cholangitis, an immune-mediated cholestatic disease (15). Conversely, type I IFNs can protect against TLR-9-induced liver injury and promote the production of IL-1 receptor antagonist, countering IL-1β (16). Notably, Type I IFNs can suppress liver disease progression (17), with IFN-α treatment alleviating fibrosis in chronic hepatitis C biopsy specimens (18) and reducing serum markers of fibrosis (19). However, the molecular mechanism of type I IFN-mediated regulation of liver disease is unclear.

Macrophages have been reported to play a distinct role in resolving fibrosis by promoting ECM degradation through increased expression of multiple metalloproteinases (MMPs) (20). Macrophages play an important role in the response to tissue injury and in wound healing as a heterogeneous population of cells. Kupffer cells (KCs) are liver-resident macrophages that originate from embryonic yolk sac precursors. In addition, bone marrow-derived circulating monocytes are recruited to sites of tissue injury and infiltrating macrophages arising from *in situ* differentiation of recruited monocytes can be considered a type of liver macrophage (21). Macrophages are classified into two phenotypes: M1 and M2 (22). M1 macrophages are associated with proinflammatory response and favor Th1 responses, producing inflammatory cytokines. M2 macrophages are involved in tissue remodeling, immunomodulation, and allergies (23). Hepatic macrophages play a crucial role in liver fibrosis. KCs are activated to produce pro-inflammatory cytokines that promote the inflammatory response and participate in liver fibrogenesis by recruiting monocyte-derived macrophages to the liver through CCL2 and CCL5 (24).

Macrophages play dual roles in tissue damage and repair. In cases of hepatic fibrosis regression, blocking harmful processes can halt and partially reverse fibrosis progression (21). Several macrophage-derived MMPs involved in this process have been identified (20). Additionally, activated Kupffer cells (KCs) contribute to liver fibrosis regression by inducing hepatic stellate cell death via caspase-9 and receptor signaling mechanisms (25). However, the link between innate and adaptive immunity in liver fibrosis remains largely unexplored, aside from the involvement of type I interferon (IFN) signaling and some functions mediated by KCs. In this study, we examined the role of macrophage mediators in liver fibrosis using CCl4-treated mice, specifically investigating how IFNAR1 signaling regulates fibrosis. Our findings indicate that macrophage-derived STAT-3 signaling protects against liver fibrosis through type I IFNs.

## 2 Materials and methods

### 2.1 Animal study design

**Figure 1:**
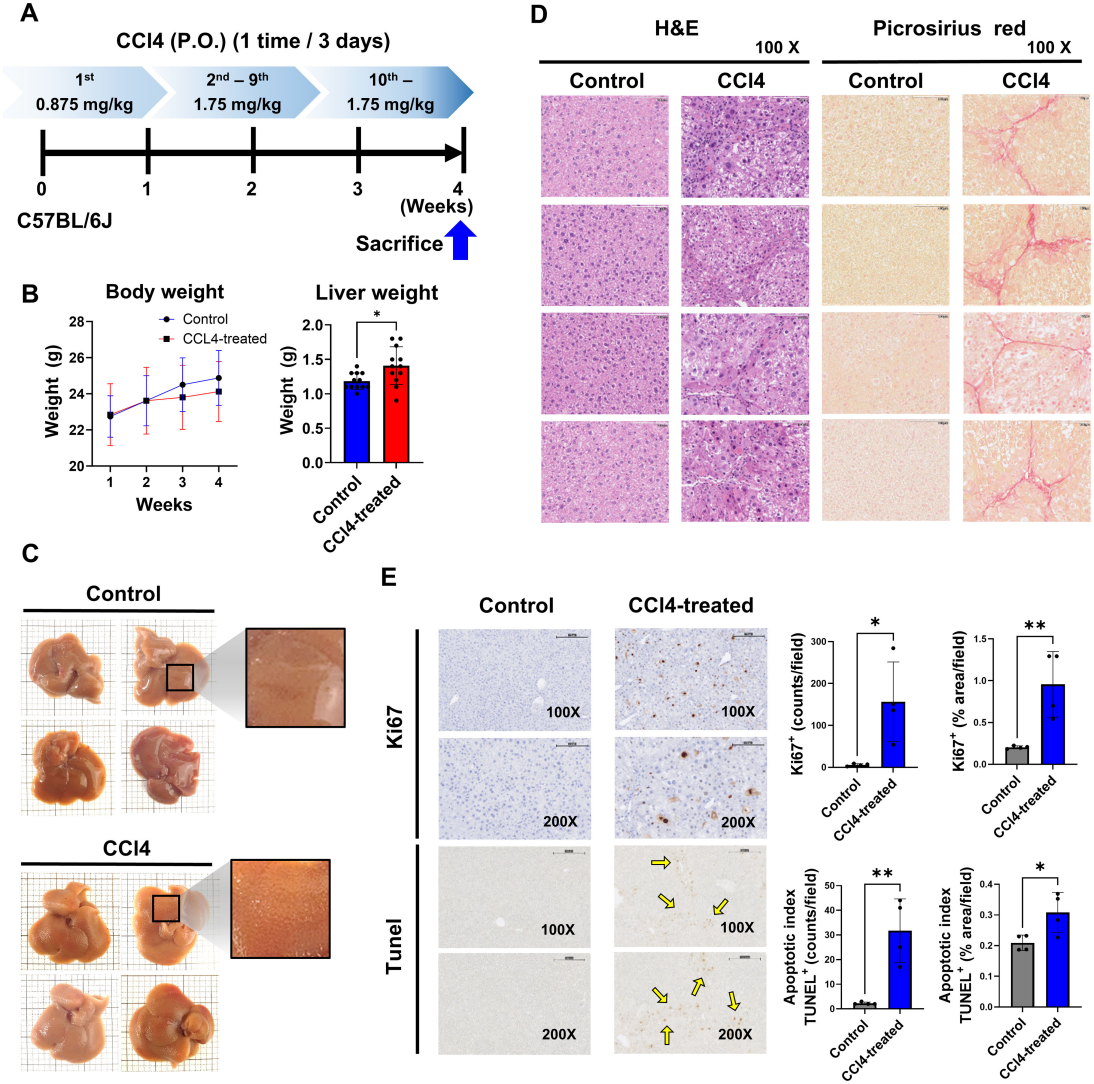
Liver fibrosis impacts cell proliferation and apoptosis. Mice in the CCl4 group received escalating doses of CCl4 every 3 days via oral gavage for 4 weeks (1^st^ dose, 0.875 mL/kg; 2^nd^ to 9^th^ dose, 1.75 mL/kg; 10^th^ to 12^th^ dose, 2.5 mL/kg). The liver tissues were analyzed for fibrosis by histological studies and status of cell proliferation and apoptosis. **(A)** Experimental design, **(B)** Body weight and liver weight (n=12), **(C)** Observed liver surface change (n=4), **(D)** H&E staining and Picrosirius red staining analysis (n=4), **(E)** Quantification of cell proliferation and apoptosis by Ki67 and TUNEL assay (n=4). Data represent the mean ± SE from three independent experiments. *p < 0.05, **p < 0.01.

**Figure 2:**
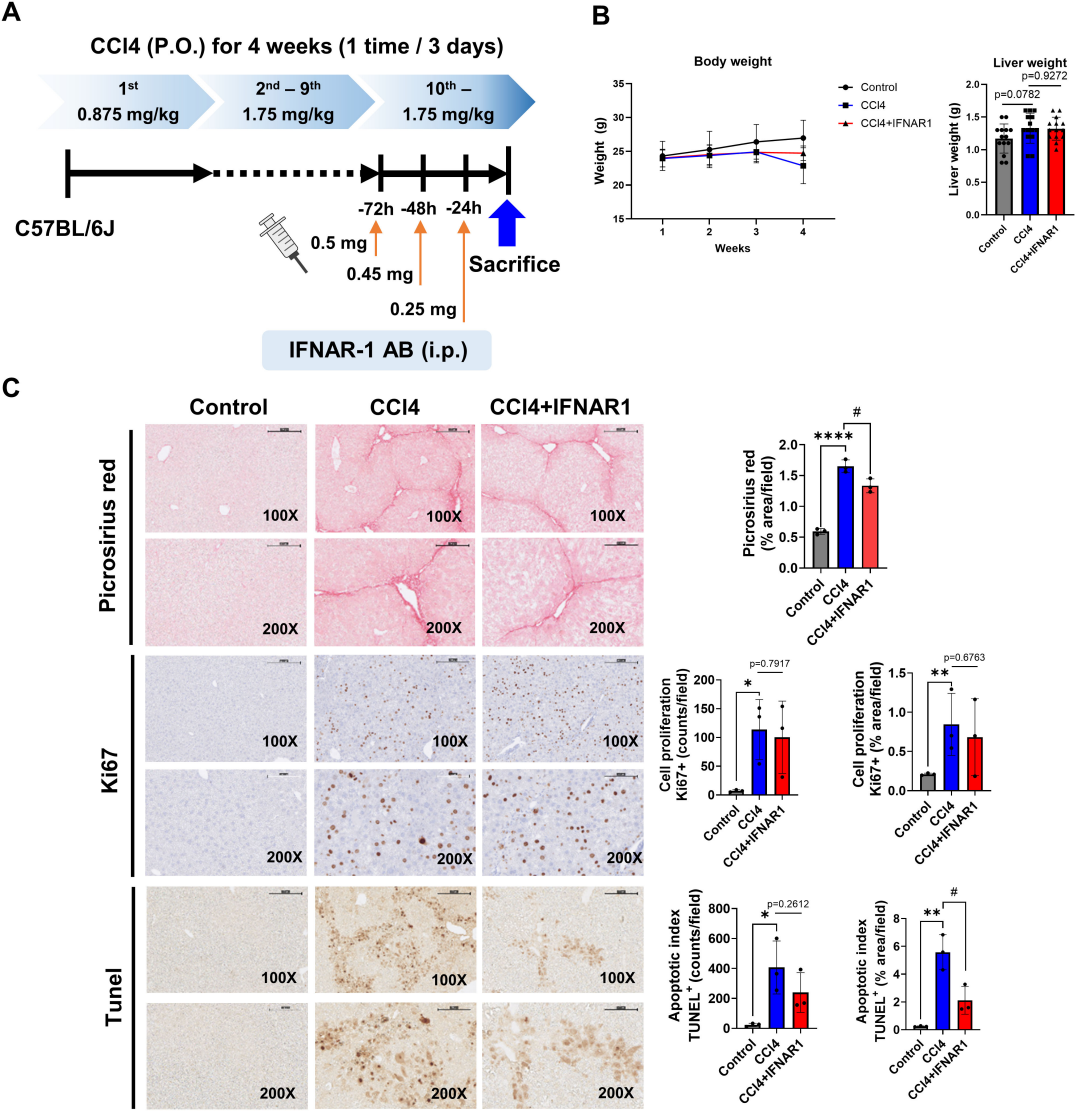
IFNAR1 blockade can improve liver fibrosis, cell proliferation, and apoptosis in CCl4-treated mice. Mice in the CCl4 group received escalating doses of CCL4 every 3 days via oral gavage for 4 weeks (1^st^ dose, 0.875 mL/kg; 2^nd^ to 9^th^ dose, 1.75 mL/kg; 10^th^ to 12^th^ dose, 2.5 mL/kg). Mice in the CCl4+IFNAR1 group were injected with Anti-Mouse IFNAR-1 three times (0.5 mg/kg, 0.45 mg/kg, and 0.25 mg/kg at 72 h, 48h, and 24 h before sacrifice, respectively). **(A)** Experimental design, **(B)** Body weight and liver weight (n=4), **(C)** Picrosirius red staining (n=3), Ki67 staining (n=3), and TUNEL assay (n=3). Data represent the mean ± SE from three independent experiments. *p < 0.05, **p < 0.01, ****p < 0.0001, ^#^p < 0.05.

Male C57BL/6J mice (6-8 weeks old) were purchased from the Jackson Laboratory (Bar Harbor, ME, USA). Mice were group housed in cages of up to four or five and maintained under a 12-hours light/dark cycle with ad libitum access to food and water. The mice were randomly divided into two groups (control and CCl4 groups) in the preliminary animal experiment, and into three groups (control, CCl4, and CCl4+IFNAR1 groups) in the main experiment. Mice in the CCl4 and CCl4+IFNAR1 groups were administered with CCl4 (carbon tetrachloride, Sigma-Aldrich, St Louis, MO, USA) every three days via oral gavage, receiving escalating doses over time (first dose: 0.875 mL/kg; second to ninth dose: 1.75 mL/kg; tenth to twelfth dose: 2.5 mL/kg) for four weeks (**Figures 1A**, **2A**). The mice in the CCl4+IFNAR1 group were injected with Anti-Mouse IFNAR-1 Purified *in vivo* GOLD™ Functional Grade (Leinco Technologies, Inc. Fenton, MO, USA) three times (0.5 mg/kg, 0.45 mg/kg, and 0.25 mg/kg at 72 hours, 48 hours, and 24 hours before sacrifice, respectively) (**Figure 2A**). According to the manufacturer’s datasheet, IFNAR-1 is classified as a type I membrane protein and is also referred to as the interferon-α/β receptor α-chain precursor. Additionally, the background information states that IFNAR1 functions as a type I membrane protein that binds to all type I IFNs, including IFN-α and IFN-β. The mice were anesthetized by intraperitoneal (i.p.) injection of avertin (250 mg/kg, 2,2,2-tribromoethanol, Sigma-Aldrich). The liver was perfused with an enzymatic solution containing 1X HBSS without Ca & Mg (Gibco, Grand Island, NY, USA), 0.01% collagenase IV (Sigma-Aldrich), and 0.05% fatty acid-free BSA (Sigma-Aldrich). Liver tissue was collected for liver cell isolation, histological and immunohistochemical analysis, and RNA or protein extraction. All mice used in this experiment were maintained at the animal facility of the University of Virginia School of Medicine. This study was approved by the Institutional Animal Care and Use Committee (IACUC) of the University of Virginia School of Medicine. All experimental methods were conducted in accordance with animal care guidelines and regulations and were approved by the ethics committee of the University of Virginia School of Medicine.

### 2.2 Isolation of mouse non-parenchymal liver cells

Liver cells were isolated using the two-step collagenase perfusion method. Liver tissue was minced with forceps to <2 mm pieces, transferred to a pre-warmed EGTA buffer containing HBSS, 0.5 mM EGTA, and 0.5% fatty acid free BSA, and agitated at 100 rpm for 10 min at 37°C. Tissue was washed with HBSS (80 × g, 10 min, 4°C), transferred to a pre-warmed digestion buffer containing HBSS, 0.05% collagenase IV, and 0.5% fatty acid free BSA, agitated at 100 rpm for 30 min at 37°C, then passed through a 100 μm cell strainer (Life Sciences, Durham, NC, USA). To remove the parenchymal cells such as hepatocytes and erythrocytes, cells were centrifuged at 50 × g for 20 sec at 4°C and washed with HBSS at 80 × g for 10 min at 4°C. Cell pellets were suspended in a 40% Percoll solution (Sigma-Aldrich) in HBSS, then gently overlaid onto a 70% Percoll solution and centrifuged at 800 × g for 25 min. NPCs were collected from the interface layer and washed twice with PBS. NPCs were counted and used for flow cytometry and F4/80 labeling.

### 2.3 Immunomagnetic labeling for F4/80+ KC

For magnetic labeling, the NPCs suspension containing buffer including the PBS, 0.5% BSA, pH 7.2, and 2 mM EDTA was centrifuged at 300 × g for 10 min. The cell pellet was resuspended in 400 μL of buffer, 100 μL of Anti-F4/80 MicroBeads UltraPure (Miltenyi Biotec, Bergisch Gladbach, Germany) was added per 10^8^ cells, mixed, and incubated at 4°C for 10 min in the dark. After labeling, the cells were washed by adding 10 mL of buffer per 10^8^ cells, centrifuged at 300 × g for 10 min, and the pellets were resuspended to a final concentration of 10^8^ cells in 500 μL of buffer.

For magnetic separation, the LS column was inserted into the MACS separator. The column was rinsed with 3 mL of buffer and allowed to run through without bubble. The labeled cell suspension was applied onto the column, and flow-through containing unlabeled cells was collected. The column was washed 3 times with 3 mL of buffer. Unlabeled cells that passed were collected again. The column was removed from the separator and placed in another collection tube. To collect the labeled cells, 5 mL of buffer was applied onto the column, and flushed out the magnetically labeled cells by firmly pushing the plunger into the column. The F4/80+ KC cells were counted, used for RNA isolation and bulk RNA sequencing.

### 2.4 Histological analysis

Liver tissues were fixed in 10% (v/v) formalin for 48 hours, transferred to 70% ethanol, and 5-μm-thick paraffin sections were prepared for Hematoxylin and Eosin (H&E) staining and Picrosirius red staining. For H&E staining, slides were incubated with hematoxylin solution for 10 min, placed in water for washing, and then incubated with eosin solution for 3 min before being deparaffinized in xylen and rehydrated with ethanol. For Picrosirius red staining, slides were stained in sirius red working solution for 1 hour, washed, and dehydrated by immersion in ethanol and xylene solutions. All slides for H&E and Picrosirius red staining were mounted using ProLong TM Gold antifade reagent (Thermo Fisher Scientific, Waltham, MA, USA). The slides were observed under an Olympus BX40F4 microscope (Southern Microscope, Inc., Haw River, NC, USA).

### 2.5 Immunohistochemical analysis

Liver tissues were fixed in 10% (v/v) formalin for 48 hours, transferred to 70% ethanol, and 5-μm-thick paraffin sections were prepared using Plus Microscope slides (Thermo Fisher Scientific). TUNEL staining of the liver was conducted using the TUNEL Assay Kit (Abcam, Cambridge, MA, USA), according to the manufacturer’s instructions. The slides were subjected to rehydration with xylene and ethanol, permeabilization with proteinase K, quenching with 30% H_2_O_2_, equilibration with TdT Equilibration Buffer, labeling with TdT Enzyme and Labeling Reaction Mix, termination of labeling, blocking, detection, development with DAB solution, and counterstaining. Ki67 staining was performed using the robotic platform (Ventana Discovery Ultra Staining Module, Ventana, Tucson, AZ, USA) at the University of Virginia Biorepository and Tissue Research Facility. Slides were deparaffinized and a heat-induced antigen retrieval protocol was performed. The slides were blocked with a peroxidase inhibitor, incubated with a Ki67 antibody (Abcam) for 60 min at room temperature, and detected. All slides were counterstained, dehydrated, cleared, and mounted for analysis (26), then observed under an Olympus BX40F4 microscope (Southern Microscope).

### 2.6 RNA extraction and quantitative real-time reverse transcription polymerase chain reaction analysis of liver tissues

A portion of the liver was homogenized in the QIAzol^®^ Lysis Reagent (Qiagen, Valencia, CA, USA) using an IKA^®^ T10 Basic homogenizer (IKA Works, Inc., Wilmington, NC, USA). After incubating the homogenized tissue at room temperature for 5 min, 0.2 mL of chloroform (Sigma-aldrich) was added to each tube and vortexed vigorously for 15 sec, the tubes were incubated at room temperature for 3 min, and centrifuged at 12,000 × g for 15 min at 4°C. The supernatant was transferred to a new tube, and 0.5 mL of isopropanol (Sigma-Aldrich) was added to each tube, mixed thoroughly, incubated at room temperature for 10 min, and centrifuged at 12,000 × g for 10 min at 4°C. The supernatant was discarded, and 75% ethanol was added to the pellet, then centrifuged at 7500 × g for 5 min at 4°C. The supernatant was completely aspirated, and RNase-free water was added to each tube, mixed, and kept on ice before RNA determination. RNA concentration and quality assessment were performed using a Nanodrop 2000 (Thermo Fisher Scientific). Only RNA samples with a 260/280 nm ratio of 1.8–2.1 were selected for complementary DNA (cDNA) synthesis. The High-Capacity cDNA Reverse Transcription Kits (Thermo Fisher Scientific) was used for cDNA synthesis, and RT-qPCR (QuantStudio 6 Flex, Applied Biosystems, Foster City, CA, USA) was performed on samples using 1 μL of cDNA with PowerUp™ SYBR™ Green Master Mix (Applied Biosystem). The cDNA was amplified with 40 cycles of denaturation (95°C for 15 sec), annealing (56–60°C for 30 sec), and extension (70°C for 30 sec). All data were automatically calculated as delta-delta (ΔΔ)-CT values using QuantStudio™ Real-Time PCR Software v1.7.1. The sequences of mouse primers are listed in **Table 1**.

**Table 1:** Mouse primer sequences for RT-qPCR.

Gene	Sequence (forward)	Sequence (reverse)
*HPRT*	5’-GTGTTCTAGTCCTGTGGCCA-3’	5’-TCAAAAGTCTGGGGACGCAG-3’
*IFNα*	5’-CTGCTGGCTGTGAGGACATA-3’	5’-AGGAAGAGAGGGCTCTCCAG-3’
*IFNβ*	5’-TTCGGAAATGTCAGGAGCTCC-3’	5’-TCCGCCTCTGATGCTTAAAGG-3’
*ISG15*	5’-AAAGGTGAAGATGCTGGGGG-3’	5’-AAAGCCGGCACACCAATCTT-3’
*USP18*	5’-GAGATGTTTCGTCCAGCCCA-3’	5’-GGTTTGGGGCAGATGAGTCA-3’
*Ifi44*	5’-TCTGTGTTCAAGGGCAGCAT-3’	5’-AGGGGGTCACTGTCATCCTT-3’
*Ifit1*	5’-AAGGTGGAGAAGGTGTGCAA-3’	5’-TGCACATTGTCCTGCCTTCT-3’
*Ifit2*	5’-TGAAGCTTGACGCGGTACAT-3’	5’-AGCCTTGTCTTGACGCTTCA-3’
*IRF3*	5’-AACAACTGCCAAGCCCCAAT-3’	5’-ATTTCCCCCATGCAGAACCA-3’
*IRF7*	5’-AAGGTGTACGAACTTAGCCGG-3’	5’-AAGCGTCTCTGTGTAGTGCA-3’
*Arginase*	5’-TCGTGTACATTGGCTTGCGA-3’	5’-TGTCTGCTTTGCTGTGATGC-3’
*Lipoxygenase*	5’-TTGCTGCACTTTGGTCCTGA-3’	5’-GGCTGCGTCATTTGGGTAAT-3’
*IL-6*	5’-CCACGGCCTTCCCTACTTC-3’	5’-TGGGGAGTGGTATCCTCTGTGA-3’
*IL-1β*	5’-AGTTGACGGACCCCAAAAGA-3’	5’-GGACAGCCCAGGTCAAAGG-3’
*TNF-α*	5’-CACAAGATGCTGGGACAGTGA-3’	5’-TCCTTGATGGTGGTGCATGA-3’

### 2.7 Western blotting

A portion of the liver was homogenized in Pierce™ RIPA Buffer (Thermo Fisher Scientific) with Halt™ Protease and Phosphate Inhibitor Cocktail (Thermo Fisher Scientific) using an IKA^®^ T10 Basic homogenizer (IKA Works, Inc.). The homogenized liver samples were placed on ice and vortexed every 10 min for 1 hour, and centrifuged at 12,000 × g for 20 min at 4°C. The supernatant containing protein was transferred to a new tube, and the protein concentration of lysates was determined using Bio-Rad Protein Assay Dye Reagent Concentrate (Bio-Rad Laboratories, Hercules, CA, USA). Protein samples (20–80 μg) were loaded into 4–15% Mini-PROTEAN TGX Gels (Bio-Rad), and transferred using Trans-Blot Turbo Transfer Pack (Bio-Rad) and Trans-Blot^®^ Turbo™ Transfer system (Bio-Rad). The transferred membrane was blocked with a blocking buffer containing 5% skim milk (Bio-Rad) in Tris-buffered saline (TBS, Bio-Rad) with 1% Triton^®^ X-100 (Sigma-Aldrich) for 1 hour at room temperature. The membranes were washed, and incubated overnight at 4°C with primary antibodies recognizing either β-actin (Cell Signaling Technology, 1:1000), IFNβ (Invitrogen, 1:1000), ISG15 (Invitrogen 1:1000), USP18 (Invitrogen 1:1000), phopho-STAT1 (Cell Signaling Technology, 1:1000), STAT1 (Cell Signaling Technology, 1:1000), phospho-STAT3 (Cell Signaling Technology, 1:1000), STAT3 (Cell Signaling Technology, 1:1000), Arginase-1 (Cell Signaling Technology, 1:1000), and Alox12 (Cell Signaling Technology, 1:1000). The membranes were washed three times, and incubated with horseradish peroxidase-conjugated secondary antibodies (Cell Signaling Technology, 1:2000) for 1 hour at room temperature. The membranes were washed three times, visualized with SuperSignalTM West Femto Maximum Sensitivity Substrate (Thermo Fisher Scientific) using AMERSHAM ImageQuant 800 (Cytiva, Marlborough, MA, USA), and analyzed using ImageJ software (ImageJ 2.1.0, USA).

### 2.8 Flow cytometry

The following mouse antibodies were used for cell surface and intracellular staining: anti-CD45 (FITC, BD Biosciences, Franklin Lakes, NJ, USA), anti-CD45 (APC, Invitrogen), anti-CD3e (ACP-eFluor780, Invitrogen), anti-NK1.1 (PE-Cy7 or eFluor660, eBioscience, San Diego, CA, USA), anti-CD4 (AF700, Invitrogen), anti-CD8a (PE or PerCP-eFluor710, eBioscience), anti-CD44 (PE-Cy7, eBioscience), anti-KLRG1 (PerCP-eFluor710, eBioscience), anti-CD69 (APC or FITC, BioLegend, San Diego, CA, USA), anti-CD11b (PerCP-Cy5.5, BD Biosciences), anti-F4/80 (PE or FITC, eBioscience), anti-CD11c (FITC, Invitrogen), anti-CD11c (PE, BD Biosciences), anti-CD19 (PE-Cy7, Invitrogen), anti-CD19 (PE, eBioscience), anti-CD86 (PECy7, Invitrogen), anti-CD206 (AF700, Invitrogen), anti-P-STAT1 (AF647, BioLegend), and anti-P-STAT3 (BV421, BioLegend). Live and dead cells were identified using the CellTraceTM Violet Cell Proliferation Kit (Thermo Fisher Scientific) and the Aqua Live/Dead Fixable Dead Cell stain Kit (Invitrogen).

Separated NPCs from mouse liver tissue were washed and dispensed into tubes at 3 × 10^6^ cells/tube. For cell surface staining, NPCs were incubated with a single antibody or an antibody cocktail according to the manufacturer’s instruction for 30 min and then washed. For intracellular staining, NPCs were fixed with 500 μL of fixation buffer (Biolegend) per 10^6^ cells at 37°C for 15 min, permed with 1 mL of Perm buffer (BD Biosciences) per 10^6^ cells, and incubated with a single antibody or antibody cocktail for 30 min, with washing between steps. Stained cells were suspended in Cell Staining Buffer (BioLegend) and analyzed using Attune NxT Acoustic Focusi Cytometer (Invitrogen).

### 2.9 RNA extraction and Single cell RNA-seq of F4/80+ KC

The RNA extraction for immunomagnetic-labeled F4/80^+^ KC was performed by combining conventional methods with the RNeasy^®^ Mini Kit (Qiagen) method. The F4/80^+^ KCs, as a single-cell type, were homogenized in the QIAzol^®^ Lysis Reagent (Qiagen) using an IKA^®^ T10 Basic homogenizer (IKA Works, Inc.). After incubating the homogenized tissue at room temperature for 5 min, 0.2 mL of chloroform (Sigma-aldrich) was added to each tube, vortexed vigorously for 15 sec, incubated at room temperature for 3 min, and centrifuged at 12,000 × g for 15 min at 4°C. The supernatant was collected and 0.5 mL of isopropanol (Sigma-Aldrich) was added, mixed thoroughly, incubated at room temperature for 10 min, and centrifuged at 12,000 × g for 10 min at 4°C. The supernatant was discarded, 1 mL 70% ethanol was added to the pellet, and mixed well by pipetting. The proper volume (700 μL) of the sample was transferred to an RNeasy Mini spin column (Qiagen) in a 2 mL collection tube (Qiagen), centrifuged for 15 sec at 8000 × g, and the flow-through was discarded. The Buffer RW1 (700 μL) and Buffer RPE (500 μL, twice) were used for washing, and centrifuged for 2 min at 8000 × g in the final washing step. The centrifuged RNeasy spin column was placed in a new 2 mL collection tube, and centrifuged for 1 min at maximum speed to dry. The RNeasy spin column was then transferred to a new 1.5 mL collection tube, and the RNase-free water (25 μL) was added, centrifuged 1 min at 8000 × g, and kept on ice for RNA determination. RNA concentration and quality assessment were performed using a Nanodrop 2000 (Thermo Fisher Scientific).

For scRNA-seq analysis, RNA sample quality testing, library construction, quality control, and sequencing were performed. During data quality control, clean data were obtained by removing all reads including adapter, reads with ploy-N sequences, and low-quality reads from the raw data. The reference genome was built using HISAT2 v2.0.5 (27), which generates a database of splice junctions. Quantification of gene expression levels was performed using featureCounts v1.5.0-p3 (28), which counts the number of reads mapped to each gene. The expected number of fragment per kilobase of transcript sequence per millions base pairs sequenced (FPKM) for each gene was calculated based on gene length and the number of mapped reads. Differential expression analysis of two biological replicates per condition was performed using the DESeq2Rpackage (1.20.0) (29). For each sequenced library, read counts were adjusted by edgeR through a scaling normalized factor. Differential expression analysis between two conditions was conducted using edgeR (3.22.5).

### 2.10 Statistical analysis

For pairwise comparisons, we primarily employed the Student’s t-test, under the assumption that the data followed a normal distribution. For data without normal distribution, we additionally performed the Mann-Whitney U test as a non-parametric alternative to validate the robustness our results. This approach ensures that statistical conclusions are not biased by deviations from normality. All statistical analyses were conducted using GraphPad Prism v10.2.2 (Boston, MA, USA), and a p-value of less than 0.05 was considered statistically significant.

## 3 Results

### 3.1 Liver fibrosis is associated with increased hepatic cell proliferation and apoptosis

To study the role of immune cells in liver fibrosis, we used a murine model treated with CCl4 via oral administration every three days, with escalating doses (0.875 mg/kg in the first week; 1.75 mg/kg in weeks two and three) (**Figure 1A**). We evaluated body weight, liver weight, and histological changes in mice for 4 weeks of treatment. The body weight of the CCl4-induced fibrosis group significantly decreased compared to the control group from 2 weeks to later weeks, while liver weight markedly increased (**Figure 1B**). The liver surface in the CCl4 group were rough and uneven, contrasting with the smooth surface in the control group (**Figure 1C**).

Liver damage was assessed by performing histology studies. H&E staining revealed a significant increase in hepatocytes and Kupffer cells in the CCl4 group compared to controls. Picrosirius red staining confirmed the presence of fibrotic regions in the portal areas (**Figure 1D**). Ki67 staining showed a notable rise in Ki67-positive liver cells in the CCl4 group, indicating increased cell proliferation. Furthermore, TUNEL-positive apoptotic cells were significantly elevated in the CCl4 group, suggesting that liver fibrosis development involves ongoing cells apoptosis and proliferation (**Figure 1E**).

### 3.2 Macrophages and monocytes are significantly elevated in liver fibrosis

**Figure 3:**
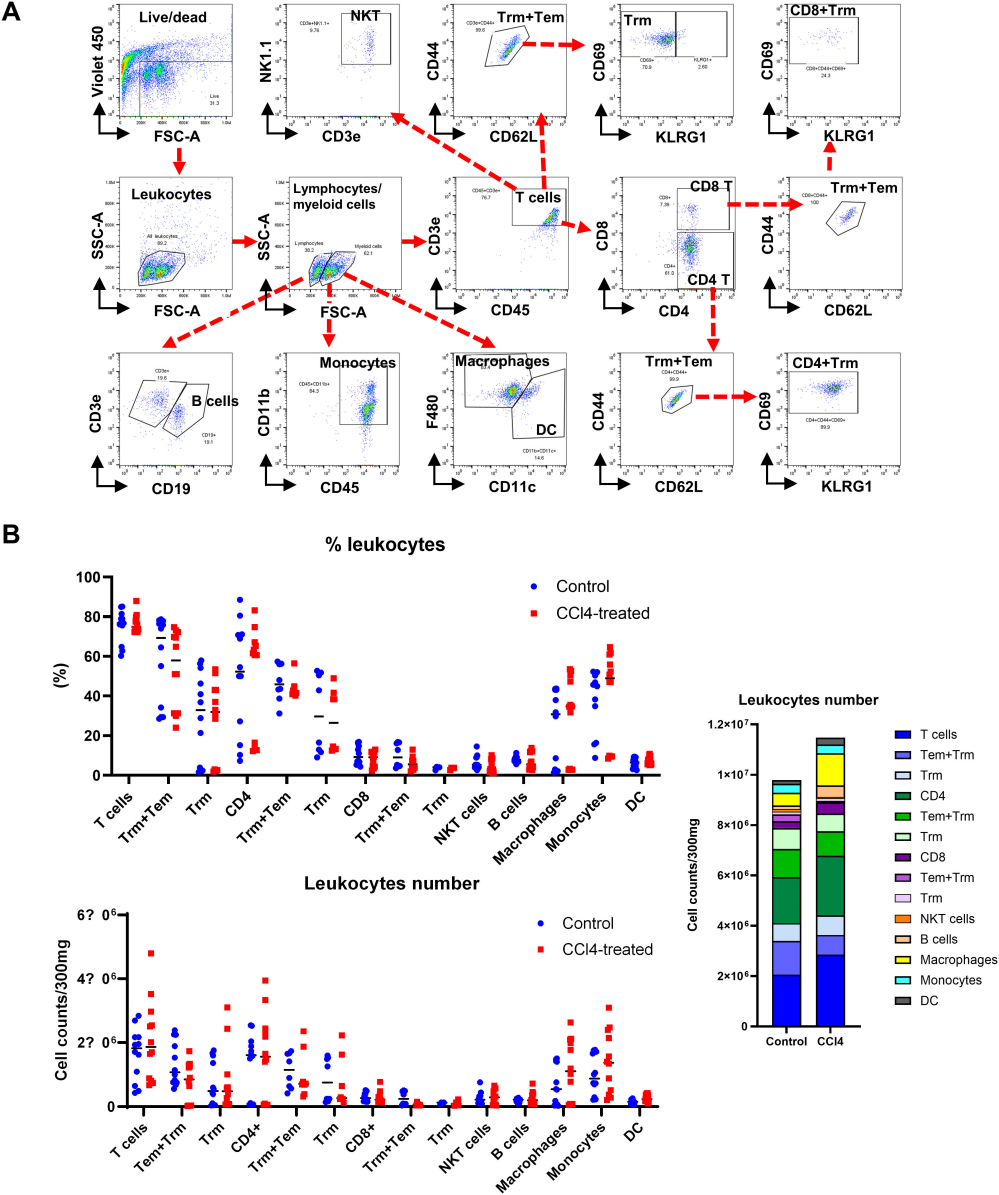
Liver fibrosis increases the number of macrophages and monocytes. Non-parenchymal liver cells (NPCs) were isolated from whole liver cells by using Percoll solution. After counting the number of NPCs, lymphoid and myeloid cell subsets in NPCs were analyzed by flow cytometry. **(A)** Flow cytometry analysis of liver leukocyte population data. **(B)** Liver leukocyte population analyzed for cell type proportions and total numbers (n=12). All comparisons between the two groups within each factor showed no significant differences (*p* > 0.05, unpaired t-test).

To assess the impact of immune cells on liver fibrosis, we analyzed liver leukocytes populations using flow cytometry in the CCl4 model. Total non-parenchymal cells (NPCs) were isolated from untreated and CCl4-treated mice after 4 weeks. Following live/dead cell separation, we gated total leukocytes, differentiating lymphocytes and myeloid cells. We identified T cells (CD3e+), B cells (CD19+), NK cells (NK1.1+), and NKT cells (CD3e+NK1.1+) within the lymphocyte population. Various T cell subsets were further analyzed: from CD3e+ gate, CD4+ T cells, CD8+ T cells, CD3e+CD44+CD69+ (T cell+Trm+), CD3e+CD4+CD44+CD69+ (CD4 Tcells+Trm+), and CD3e+CD8+CD44+CD69+ (CD8 T cells+Trm+) were confirmed. Macrophages (F4/80+), monocytes (CD11b+), and dendritic cells (DC, CD11c+) were identified from myeloid cells (**Figure 3A**).

Changes in lymphocyte subpopulations were pronounced than in overall leukocyte counts (**Figure 3B**). CD4 T+ cell numbers significantly increased in fibrotic mice, while CD8+ Tcell counts remained unchanged. B cell numbers were significantly elevated in fibrotic group, yet the proportion remained similar to controls. In contrast, both the proportion and number of macrophages, monocytes, and DC were significantly higher in the CCl4-treated group (**Figure 3B**). These findings suggest a critical role for immune cells, particularly macrophages and monocytes, in liver fibrosis development. However, it is not clear how these monocytes and macrophages contribute to the liver fibrosis progression.

### 3.3 Increased expression of Type I IFN signaling genes is linked to liver fibrosis

To elucidate the underlying mechanism by which macrophages contribute to liver fibrosis progression, we investigate the impact of Type I IFN signaling on liver fibrosis. Type I IFNs link the innate and adaptive immune systems, crucial for immune response to external insults. Recognition of pathogen-associated molecular patterns (PAMPs) triggers the release of IFN-α/β, activating JAK1/2 and STAT1/2 pathways in affected cells. This signaling cascade recruits interferon-stimulated genes (ISGs). *ISG15*, a highly expressed following Type I IFN stimulation protein in this pathway, is implicated in creating an immunosuppressive tumor microenvironment (TME) and developing as immune adjuvant therapy using ISG15 targeting.

**Figure 4:**
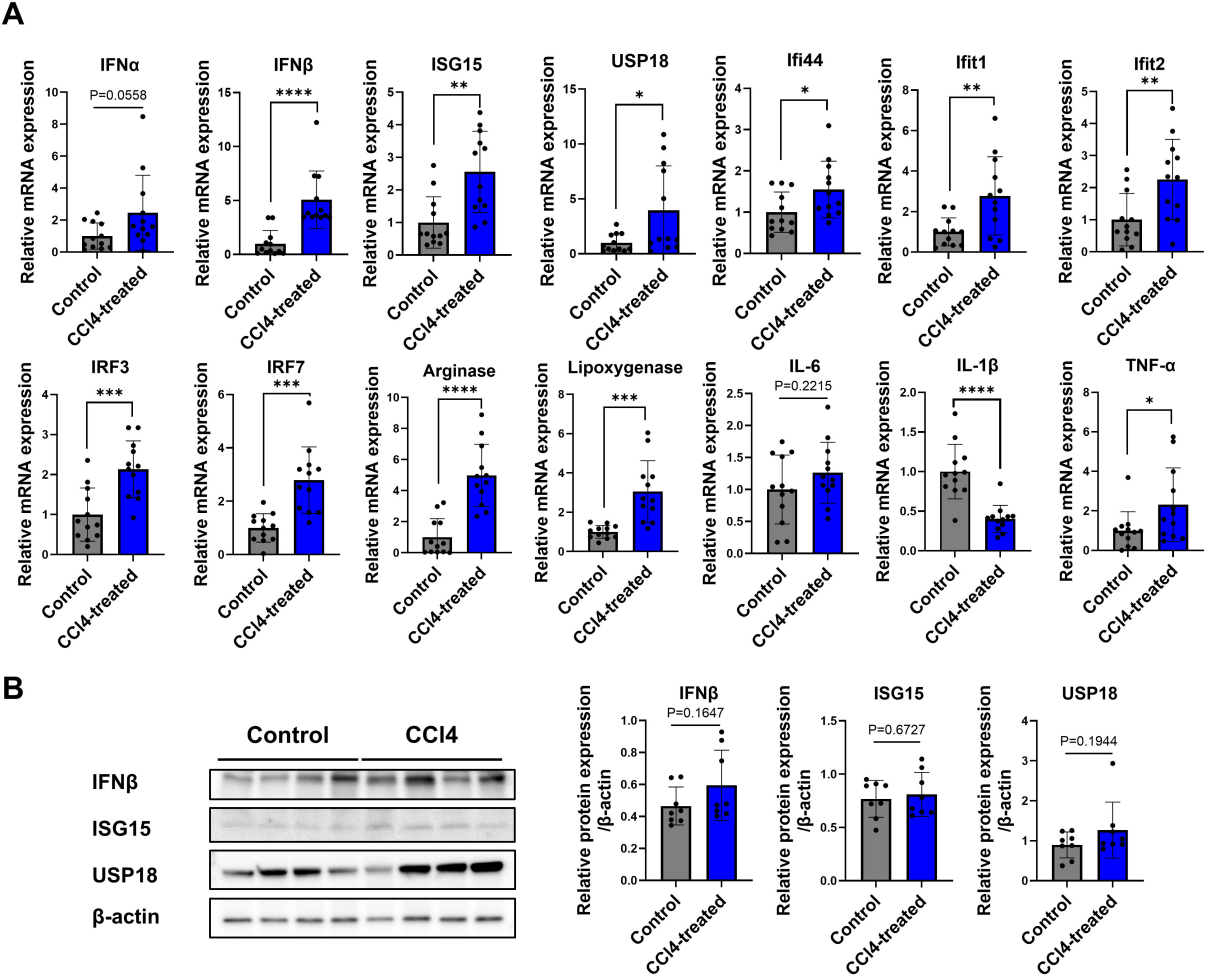
Increased expression of type I IFN signaling genes in liver fibrosis. Total RNA and proteins were extracted from whole liver tissues, real-time reverse transcription polymerase chain reaction (RT-qPCR) analysis and Western blotting were performed. **(A)** RT-qPCR expression levels for a panel of genes related to type-I IFN signaling (n=12), **(B)** Western blot of proteins related to type-I IFN signaling (n=8). Data represent the mean ± SE from three independent experiments. *p < 0.05, **p < 0.01, ***p < 0.001, ****p < 0.0001.

We assessed expression levels of IFN-stimulated gene, related cytokines, and pro-inflammatory markers. Notably, levels of *IFNα*, *IFNβ*, ISGs (including *ISG15*, *USP18*, *Ifi44*, *Ifit1*, *Ifit2*, *IRF3*, *IRF7*), and pro-inflammatory cytokines (such as *IL-1β* and *TNF-α*) were significantly elevated in the CCl4-treated group compared to control (**Figure 4A**). Increased protein levels of IFNβ, ISG15, and USP18 in the CCl4-treated group were validated by Western blot analysis (**Figure 4B**). These results indicate that type-I IFN signaling contributes to liver fibrosis development.

### 3.4 Blockade of Type I IFN receptor signaling alleviates liver fibrosis and cell apoptosis in the CCL4-treated mice

To evaluate the role of type I IFN signaling in liver fibrosis, cell proliferation and apoptosis associated with liver regeneration defects, we established an IFNAR1 blockade model by administering IFNAR1 antibodies to CCl4-treated mice for three days prior to liver harvest (**Figure 2A**) (30). Body weight in the CCl4+IFNAR1 group decreased similarly to the CCl4-only group from 1 to 3 weeks of treatment but showed a slight increase at four weeks (**Figure 2B**). In contrast, liver weight was elevated in both the CCl4 and CCl4+IFNAR1 groups due to fibrosis development (**Figure 2B**).

Picrosirius red staining revealed a significant reduction in liver fibrosis in the CCl4+IFNAR1 group compared to the CCl4-only group, both visually and quantitatively (**Figure 2C**). Ki67 staining showed a decrease in the proliferation index in the CCl4+IFNAR1 group, this was not statistically significant whereas TUNEL assay demonstrated a significant reduction in apoptotic cells in the CCl4+IFNAR1 group compared to the CCl4 group (**Figure 2C**). Overall, analysis of fibrosis severity, cell proliferation, and apoptosis suggests that IFNAR1 blockade alleviates CCl4-induced liver fibrosis by reducing both cell proliferation and apoptosis.

### 3.5 Type I IFN receptor blockade enhances cell survival and anti-inflammatory signaling in monocyte-derived macrophages during liver fibrosis

**Figure 5:**
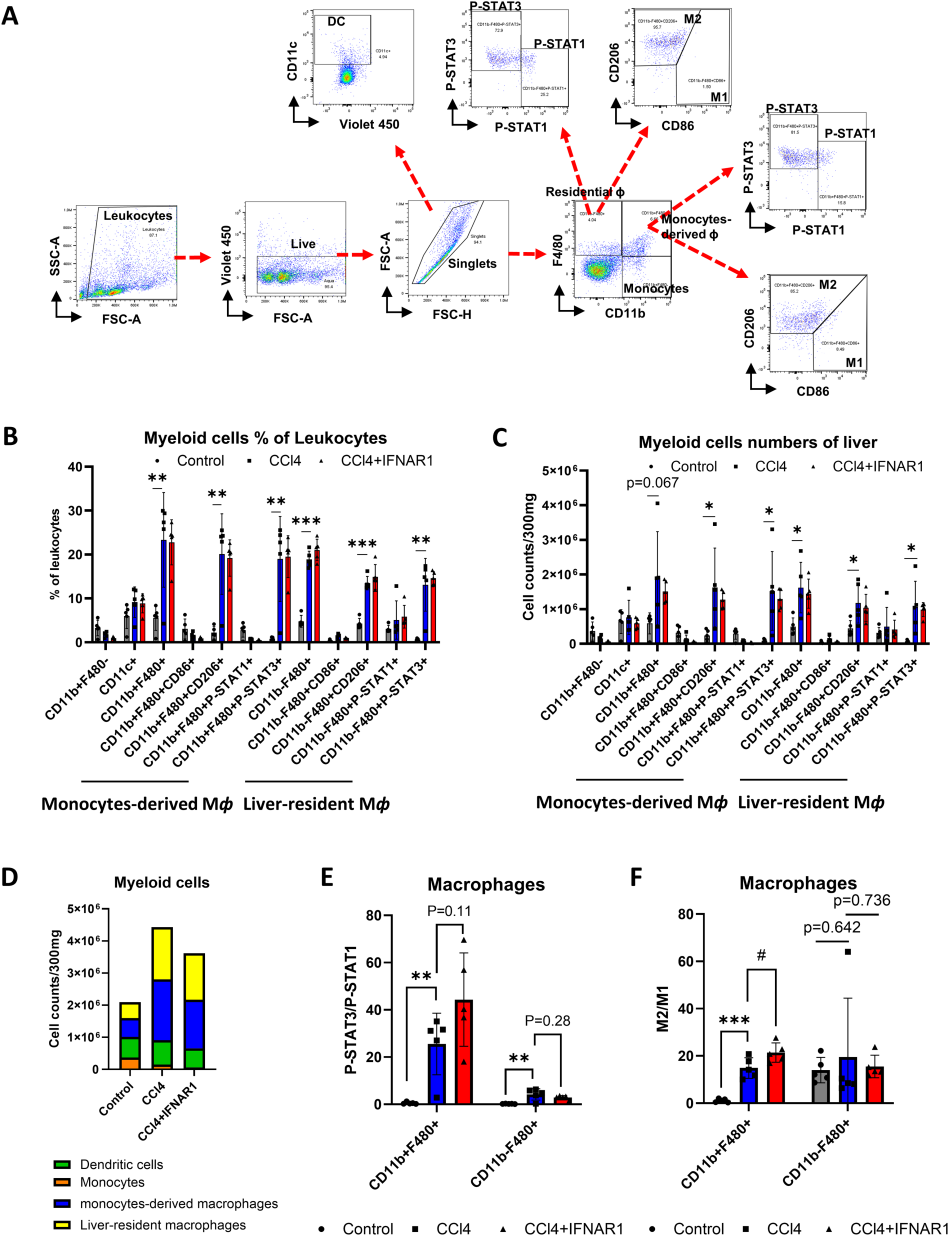
IFNAR1 blockade increases cell survival and anti-inflammatory signaling in monocytes-derived macrophages in the fibrosis mouse liver. Mice of CCl4 and CCl4+IFNAR1 groups were administered with CCl4 every 3 days via oral gavage for 4 weeks. The CCl4+IFNAR1 group were injected with Anti-Mouse IFNAR-1 3 times (0.5 mg/kg, 0.45 mg/kg, and 0.25 mg/kg at 72 h, 48h, and 24 h before sacrifice, respectively). Non-parenchymal liver cells (NPCs) were isolated form whole liver cells by using Percoll solution. **(A)** Flow cytometry analysis of liver leukocyte population data. **(B-D)** Liver leukocyte population analyzed for cell type proportions and total numbers (n=5). **(E)** The ratio of P-STAT3/P-STAT1 in the monocytes-derived macrophages and liver-resident macrophages (n=5). **(F)** Ratio of M2/M1 in monocytes-derived macrophages and liver-resident macrophages (n=5). Data represent the mean ± SE from three independent experiments. *p < 0.05, **p < 0.01, ***p < 0.001, ^#^p < 0.05.

Building on our previous analysis of liver leukocyte distribution, we next examined the function and subsets of monocyte-derived macrophages and tissue-resident macrophages in the IFNAR1 blockade model. To assess the impact of these subsets on cell survival and anti-inflammatory responses, we used flow cytometry to analyze total liver macrophages, identifying non-parenchymal cells (NPCs) by specific markers. After live/dead cell separation, we categorized macrophages (F4/80+), monocytes (CD11b+), P-STAT1, P-STAT3, M1 cells (CD86+), and M2 cells (CD206+) within the NPCs (**Figure 5A**). We then compared the frequency and numbers of myeloid cells in the NPCs among control, CCL4, and CCL4+anti-IFNAR Ab treatment groups. Both monocyte-derived and tissue resident macrophages were significantly increased in the CCl4-treated group compared to controls, with a notable rise in CD206+ and P-STAT3+ cells. In contrast, in the CCl4+IFNAR1 group, monocyte-derived macrophages showed reduced levels of CD206+ and P-STAT3+ cells, while liver-resident macrophages exhibited a further increase. Similar trends were observed in the frequency and number of myeloid cells (**Figures 5B, C**).

The total number of myeloid cells was significantly higher in the CCl4-treated group compared to controls but decreased in the CCl4+IFNAR1 group (**Figure 5D**). Analysis of the P-STAT3/P-STAT1 ratio in macrophages revealed a significant increase in both monocyte-derived and tissue resident macrophages in the CCl4-treated group, compared to controls (**Figure 5E**). The ratio of P-STAT3/P-STAT1 in monocyte-derived macrophages in the CCl4+IFNAR1 group was higher than in the CCl4-treated group with statistical significance and this difference in liver resident macrophages did not reach statistical significance (**Figure 5E**). The M2/M1 ratio in macrophages was also significantly higher the CCl4-treated group compared to controls, with statistical significance observed only in monocyte-derived macrophages (**Figure 5F**). Furthermore, the M2/M1 ratio in monocyte-derived macrophages was significantly higher in the CCl4+IFNAR1 group compared to the CCl4-treated group (**Figure 5F**).

Overall, IFNAR1 blockade enhances anti-inflammatory signaling and cell survival in monocyte-derived macrophages during CCl4-induced liver fibrosis, as evidence by increased M2/M1 ratios and P-STAT3 activation. These findings suggest that IFNAR1 inhibition may mitigate fibrosis progression via modulation of the inflammatory response toward M2 macrophage polarization.

### 3.6 scRNA-Seq of liver F4/80-positive macrophages reveals gene regulation mediated by IFNAR1 blockade

**Figure 6:**
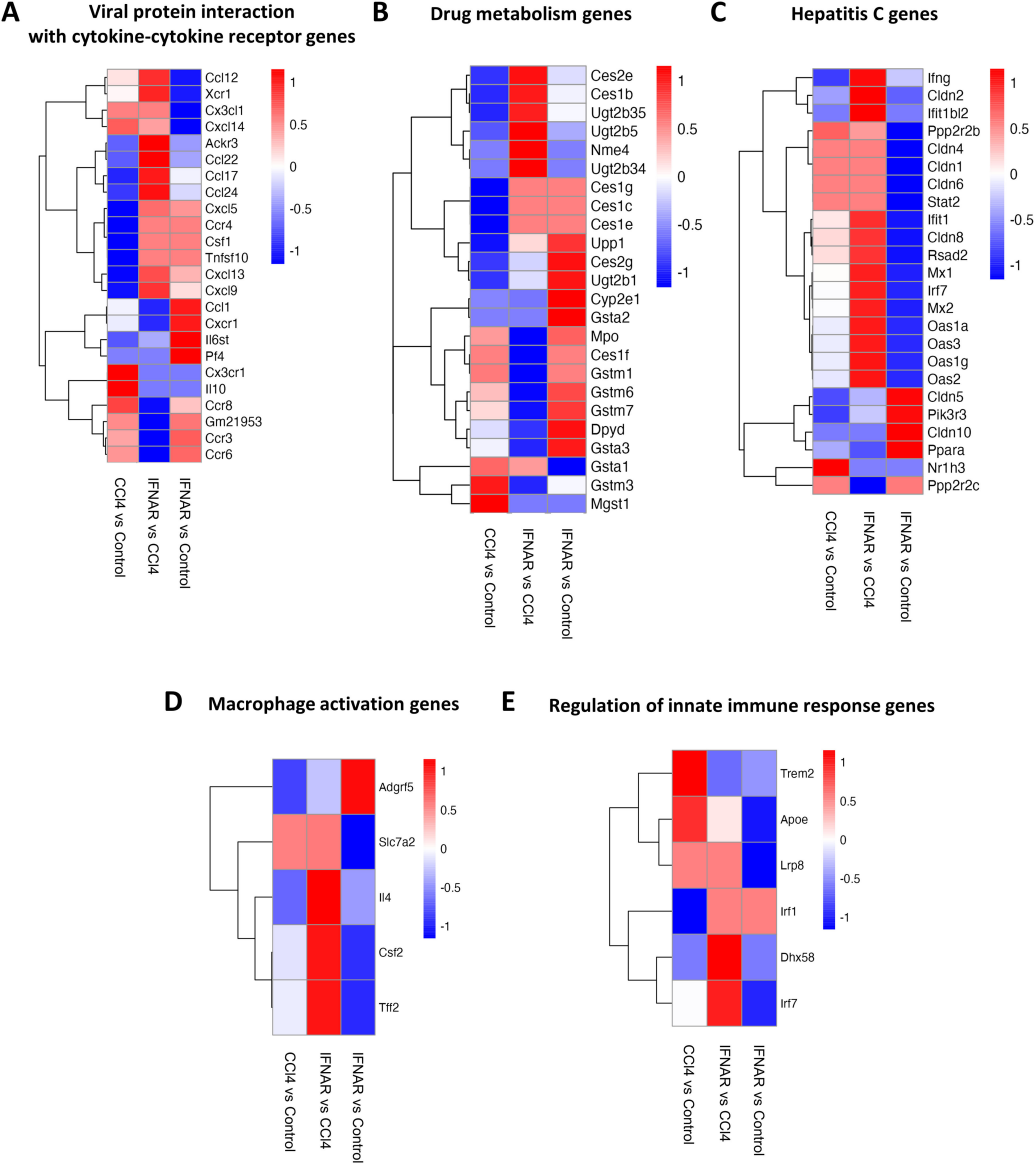
Regulation of gene expression in liver F4/80-positive macrophages by IFNAR1 blockade. Heat maps display expression of **(A)** genes involved with of viral protein interactions with cytokine-cytokine receptors, **(B)** genes involved in drug metabolism, **(C)** genes involved in hepatitis C infection, **(D)** genes involved in macrophage activation, and **(E)** genes involved in regulation of the innate immune response. Differences in gene expression are based on GSEA.

**Figure 7:**
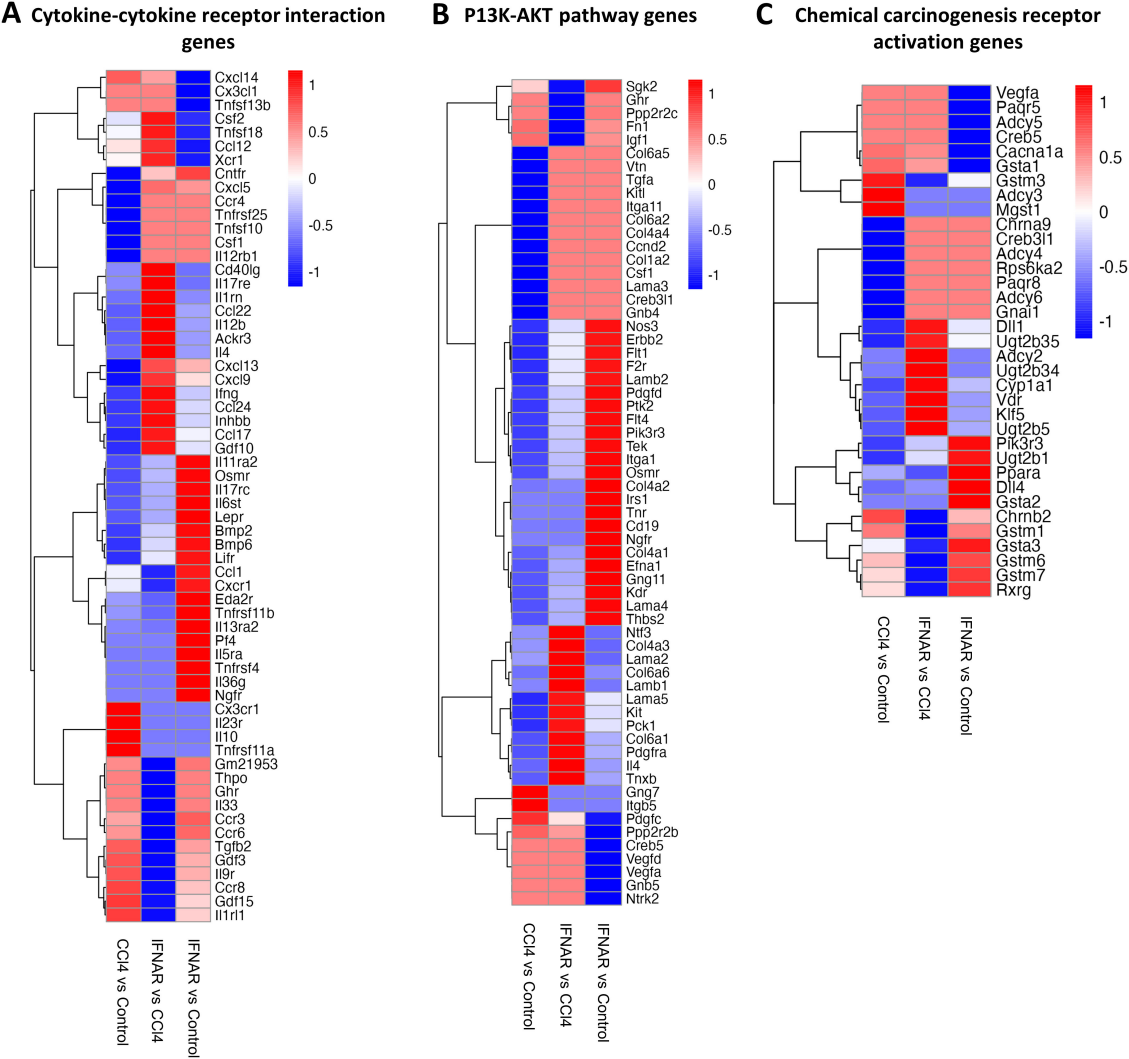
The IFNAR1 blockade modulates the expression of genes associated with various signaling pathways. Heat maps display expression of **(A)** cytokine-cytokine receptor interaction genes, **(B)** P13K-AKT pathway genes, and **(C)** chemical carcinogenesis receptor activation genes. Differences in gene expression are based on GSEA.

To assess gene expression changes in macrophages from fibrotic mice, we performed scRNA-seq on purified F4/80+ macrophages. Gen set enrichment analysis (GSEA) was used for final analysis. Results revealed significant alterations in the expression of gene associated with viral protein interactions with cytokine receptors, drug metabolism, hepatic C, macrophages activation, innate immune regulation, cytokine-cytokine receptor interaction, PI3K-AKT pathway, and chemical carcinogenesis receptor activation (**Figures 6**, **7**).

We identified several genes (*Ccl12*, *Xcr1*, *Ackr3*, *Ccl22*, *Ccl17*, *Ccl24*, *Cxcl5*, *Ccr4*, *Csf1*, *Tnfsf10*, *Cxcl13*, *Cxcl9*) that were significantly upregulated in the CCl4+IFNAR1 group compared to the CCl4-only group (**Figure 6A**). In drug metabolism, key genes (*Ces2e*, *Ces1b*, *Ugt2b35*, *Ugt2b5*, *Nme4*, *Ugt2b34*, *Ces1g*, *Ces1c*, *Ces1e*) were significantly upregulated in the CCl4+IFNAR1 group (**Figure 6B**). Notably, hepatitis C-related genes were also significantly altered, with upregulation and downregulation observed in the CCl4+IFNAR1 group (**Figure 6C**). Macrophage activation genes (*Slc7a2*, *Il4*, *Csf2*, *Tff2*) showed significant upregulation in the CCl4+IFNAR1 group (**Figure 6D**). In the regulation of innate immune response pathway, expression of Trem2 and Apoe decreased, while *Lrp8*, *Irf1*, *Dhx58*, and *Irf7* were significantly upregulated (**Figure 6E**).

Further analysis revealed a marked upregulation of cytokine-cytokine receptor interaction genes (*Csf2*, *Tnfsf18*, *Ccl12*, *Xcr1*, *Cntfr*, *Cxcl5*, *Ccr4*, *Tnfrsf25*, *Tnfsf10*, *Csf1*, *Il12rb1*, *Cd40lg*, *Il17re*, *Il1rn*, *Ccl22*, *Il1rn*, *Ccl22*, *Il12b*, *Ackr3*, *Il4*, *Cxcl13*, *Cxcl9*, *Ifng*, *Ccl24*, *Inhbb*, *Ccl17*) in the CCl4+IFNAR1 group (**Figure 7A**). Interestingly, several PI3K-AKT pathway genes (*Sgk2*, *Ghr*, *Ppp2r2c*, *Fn1*, *Igf1*, *Gng7*, *Itgb5*) were significantly downregulated (**Figure 7B**). Additionally, gene associated with chemical carcinogenesis receptor activation (*Gstm3*, *Adcy3*, *Mgst1*, *Ppara*, *Chrnb2*, *Gstm1*, *Gsta3*, *Gstm6*, *Gstm7*, *Rxrg*) were downregulated in the CCl4+IFNAR1 group (**Figure 7C**).

The scRNA-seq analysis underscores the impact of IFNAR1 blockade on gene expression in macrophages from a CCl4-induced liver fibrosis model. IFNAR1 inhibition modulated the expression of genes involved in cytokine interactions, drug metabolism, hepatitis C, macrophage activation, innate immune regulation, the PI3K-AKT pathway, and chemical carcinogenesis. Notably, key macrophage activation and anti-inflammatory signaling genes were significantly upregulated in the CCl4+IFNAR1 group, indicating differential regulation of inflammatory pathways. These findings suggest that IFNAR1 blockade plays a crucial role in promoting tissue repair and regeneration following liver injury, potentially mitigating fibrosis progression.

## 4 Discussion

In this report, we demonstrate that Type-I IFN signaling in liver macrophages plays a critical role in fibrosis development by modulating the STAT1/STAT3 pathway. Specifically, IFN blockade reduces fibrosis severity by enhancing STAT3 activation, highlighting the involvement of both STAT1 and STAT3 in tissue repair. Liver regeneration, essential for restoring tissue structure and function after injury, relies on timely cellular and molecular responses (30). This process is closely linked to inflammatory and metabolic changes involving hepatocytes, hepatic stellate cells (HSCs), and other components. KCs, the liver’s resident macrophages, contribute significantly to this regenerative process (31). Our findings underscore the role of both liver-resident and monocyte-derived macrophages in fibrosis, suggesting avenues for future research on the interactions between hepatic immune cells and liver fibrosis.

Following liver injury, the release of DAMPs or PAMPs activates KCs and monocyte-derived macrophages, which in turn release pro-inflammatory cytokines such as TNF-α and IL-1β. These cytokines exacerbate liver injury, creating a pro-inflammatory environment that disrupts hepatic homeostasis and perpetuates tissue damage. Liver injury initiates compensatory mechanisms for tissue regeneration, where hepatocytes undergo damage, proliferation, and cell death. Maintaining a balance between proliferative and inhibitory factors during these phases is essential to prevent excessive tissue damage or tumorigenesis (32). To further investigate this phenomenon, we analyzed Ki67 expression as a marker of cell proliferation. As shown in **Figure 2C**, both CCl4-treated and IFNAR-treated mice exhibited increased numbers of Tunel+ and Ki67+ cells compared to control mice. Although we did not specifically stain for resident macrophages, it is likely that some of these proliferating cells included resident macrophages, indicating their potential role in liver fibrosis.

Dysregulation of signaling pathways (Wnt/β-catenin, Hippo/Yap, HGF/c-Met, Notch, and EGFR) contributes to impaired liver regeneration, fibrosis, cirrhosis, and other liver pathologies (33). Despite the unclear role of adaptive immune cells in liver fibrosis, lymphocytes influence fibrosis progression as inhibition of lymphocyte recruitment reduces fibrosis (34). CD4+ T cells drive Th lineage polarization and cytokine production, with Th2 T cells exacerbating fibrosis by upregulation of pro-fibrotic genes and promoting of IL-10 and TGF-β in macrophages (35). While CD8^+^ T cells play a minor role, they may contribute to fibrosis by secreting pro-fibrotic cytokines (36). Tissue resident memory T (TRM) cells are involved in local immune responses and rapid reaction to recurrent infections (37, 38). Our study revealed a significant increase in CD4^+^ T cells, with a slight elevation of CD8^+^ T cells in fibrotic mice, along with an increased number of CD4^+^ TRM cells (**Figure 3**). B cell infiltration has been noted in chronic liver diseases (39, 40), yet their role in fibrosis remains uncertain. Although a trend towards increased B cell presence was observed in fibrosis, statistical significance was not reached in our studies. NKT cells, processing both pro-inflammatory and immunosuppressive roles (41), showed no significant differences between fibrotic and control mice.

Hepatic macrophages, the largest non-parenchymal cell population in the liver, play a pivotal role in inflammation and fibrosis. They are classified by origin into KCs and monocytes-derived macrophages and by phenotype into pro-inflammatory (M1) and anti-inflammatory (M2) types. These macrophages exhibit plasticity, often expressing both M1 and M2 markers simultaneously. Early liver injury is marked by pro-inflammatory macrophages, with KCs secreting IL-1β, TNF-α, CCL2, and CCL5, which activate hepatic stellate cells and recruit immune cells, promoting fibrosis (42, 43). As injury progresses, macrophages transition from pro-inflammatory to anti-inflammatory states. Some KCs adopt a wound-healing phenotype, producing matrix metalloproteinases (MMPs) that facilitate matrix degradation and fibrosis resolution (44). In later stages, anti-inflammatory macrophages predominate due to the high expression of TGF-β, emphasizing their regulatory role in fibrosis progression (45). Our study highlights the crucial involvement of monocyte-derived macrophages and KCs in fibrosis, particularly through type I IFN signaling. Since CD11b is expressed in multiple cell types, including monocytes, KCs, and other leukocytes, we distinguished monocyte-derived macrophages from KCs by comparing the expression levels of CD11b and F4/80. However, we acknowledge that the use of CD11b^+^ gating alone may lead to the detection of other leukocytes. Furthermore, Ly6C is a well-established marker for assessing the recruitment of blood monocytes to sites of liver damage. The absence of Ly6C in our analysis represents a limitation, as it may impact the accurate identification of monocyte populations. Consequently, the CD11b^+^F4/80^−^ population may include other leukocyte subsets, which should be considered when interpreting the results. Besides monocytes, CD11b is also expressed in neutrophils. While granulocytes may contribute to the progression of liver fibrosis, our study focused on analyzing liver leukocytes by gating on mononuclear cells. Therefore, the contribution of granulocytes to liver fibrosis was not assessed in this study.

Type I IFNs bridge innate and adaptive immunity, playing a key role in immune responses. Upon pathogen recognition via PAMPs, Type I IFNs-α/β activate JAK1/2, tyrosine kinase 2 (TYK2), STAT1/2, and IRF 9, leading to ISG expression (46). In this study, *IFNα*, *IFNβ*, *ISG15*, *USP18*, IFN-induced protein 44 (*Ifi44*), interferon-induced protein with tetratricopeptide repeat (*Ifit*) *1*, *Ifit2*, *IRF3*, and *IRF7* were significantly elevated in the CCl4-induced liver fibrosis model. *ISG15*, highly expressed following Type I IFN stimulation, contributes to an immunosuppressive TME and may serve as a therapeutic target (46). *USP18*, crucial in liver inflammation, correlates with poor *IFN-α* therapy outcomes in HBV and HCV patients, and its silencing enhances *IFN-α* signaling. Our study observed similar effects in fibrotic mice. *Ifi44*, first identified in HCV-infected chimpanzees (47), is upregulated specifically by Type I IFNs. Our study showed a significant increase in Ifi44 expression in the CCl4-induced liver fibrosis model, dependent on Type I IFN signaling. Ifit1 and Ifit2, key antiviral proteins, inhibit viral RNA translation and modulate RNA stability (48). Activation of Type I IFN signaling through IRF3/7 phosphorylation induces ISG transcription via the STAT1/STAT2/IRF9 complex (49). Notably, gene expression of Arginase and Lipoxygenase was significantly elevated in the CCl4-treated liver fibrosis group. Arginase metabolizes arginine, contributing to proline and polyamine production; Arginase I is induced in macrophages, whereas Arginase II is expressed in both macrophages and myofibroblasts (50). Arginase I regulates Th1 cytokines, TNF-α and IL-6 expression in a concentration-dependent manner (51). Lipoxygenases, non-hem iron-containing oxidative enzymes, regulate inflammation by producing pro-inflammatory mediators (52).

STAT3 is essential for early liver regeneration, as STAT3-deficient mice show high mortality post- hepatectomy due to increased neutrophil and monocyte infiltration, hepatocyte necrosis, and heightened inflammation (53, 54). Our study shows a significantly higher p-STAT3/p-STAT1 ratio in the liver fibrosis group, which further increases with anti-IFNAR blockade, suggesting STAT3’s role in mitigating fibrosis. STAT3, IFNs, and NF-κB are key transcription factors in macrophage polarization, with the JAK/STAT3 pathway promoting M2 polarization. Inhibiting this pathway elevates M2-promoting cytokines (IL-10 and TGF-β) while reducing pro-inflammatory cytokines (IL-6, IFN-γ, and TNF-α), thereby limiting inflammation and tissue damage (55). Our findings show that the M2/M1 macrophage ratio follows a similar trend to STAT3, reinforcing its role in M2 polarization and the antifibrotic effects of Type I IFN signaling blockade. However, since M2 macrophages can promote liver fibrosis, their effects may vary depending on the specific pathological context of liver disease.

Single-cell RNA-seq analysis confirmed the impact of type I IFN on fibrosis progression, showing reduced expression of viral protein interaction genes (*Ccl12*, *Xcr1*) and hepatitis C-related genes (*IFNg*, *Ifit1*) in the Type I IFN blockade group. Recent studies have reported that liver fibrosis stages influence the activities of organic cation transporter 1/2 (OCT1/2) in HCV-infected patients (56). Macrophage activation (*Il4*, *Csf2*) and innate immune response genes (*Dhx58*, *Irf7*) were also downregulated. Drug metabolism-related genes (*Nme4*, *Ugt2b34*) decreased, while *Mpo* and *Ces1f* increased. Additionally, fibrosis influenced cytokine receptor interaction and the PI3K-Akt pathway gene expression. In chemical carcinogenesis receptor activation, *Vegfa* and *Paqr5* were downregulated, while *Chrnb2* and *Gstm1* were upregulated. Recent studies using single-cell RNA sequencing identified HSCs and myofibroblast subsets in liver fibrosis (57). This study is significant for elucidating the role of liver macrophages in fibrosis progression with Type I IFN signaling blockade, highlighting the crucial role of Type I IFN in disease development.

## 5 Conclusion

**Figure 8:**
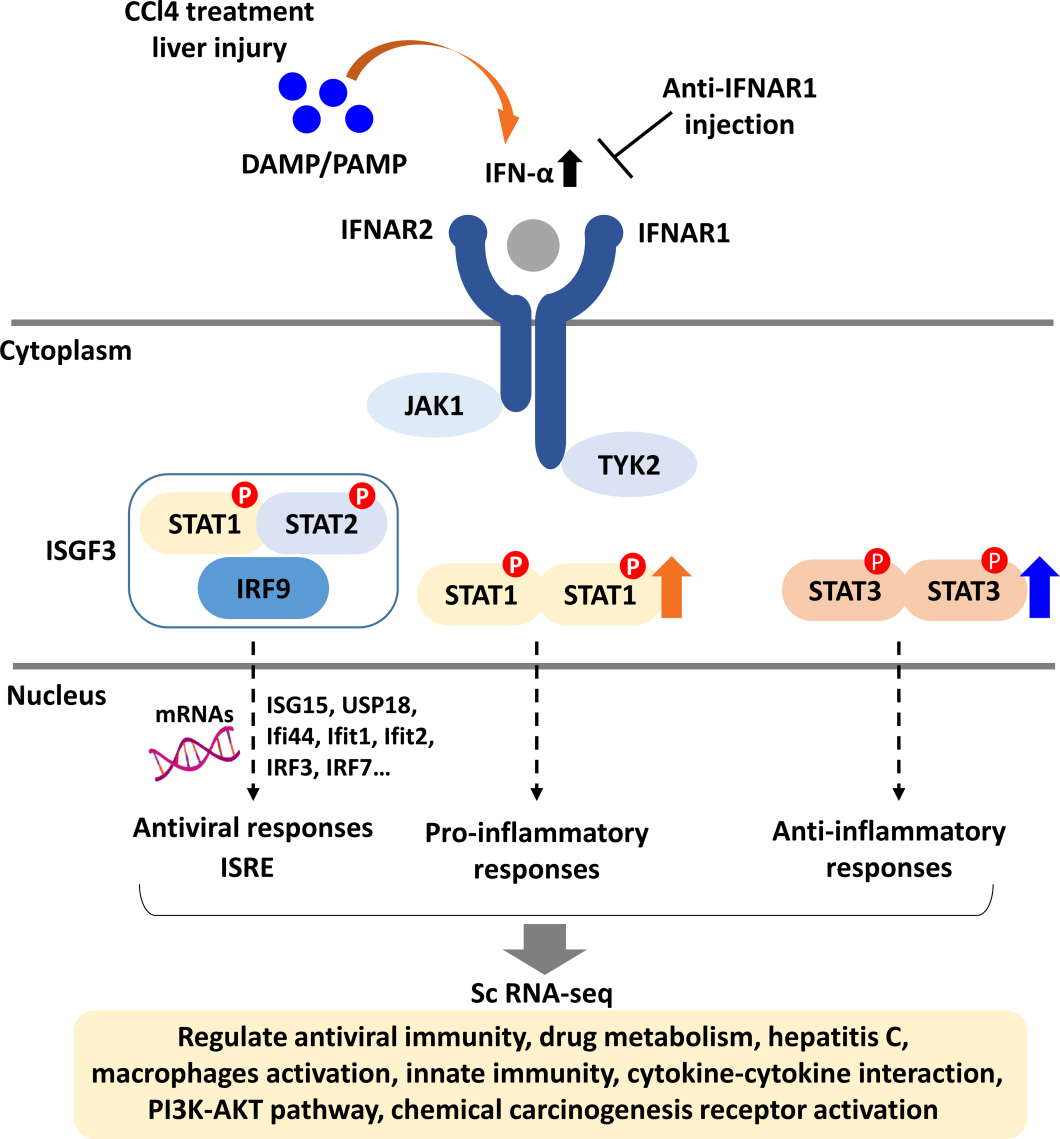
Schematic diagram of type I IFN signaling in liver fibrosis. DAMPs released from damaged liver tissue activate type I IFN signaling by triggering JAK1 and TYK2, leading to the induction of genes across three pathways: antiviral, pro-inflammatory, and anti-inflammatory responses. For the antiviral response, the IFN-stimulated gene factor 3 complex (ISGF3) binds to IFN-stimulated response elements (ISRE), promoting the expression of antiviral genes. STAT1 mediates pro-inflammatory responses, while STAT3 is involved in anti-inflammatory responses. Single-cell RNA sequencing of F4/80+ liver macrophages from fibrotic and non-fibrotic mice indicates that IFNAR1 blockade influences the expression of genes associated with various signaling pathways including innate immunity and PI3K-AKT pathway.

In conclusion, blocking Type I IFN signaling significantly alleviates liver fibrosis by modulating macrophage-derived STAT3 signaling, promoting anti-inflammatory responses, and supporting tissue repair and regeneration (**Figure 8**). The identification of various immune cells, specific myeloid cells, and gene analyses related to Type I IFN signaling through scRNA-seq collectively support these findings. This study underscores the importance of F4/80+ macrophages as key contributors to the mechanistic changes in the liver. Although this analysis may not capture the entire spectrum of liver immune cells, it highlights significant changes in monocyte-derived macrophages within the Type I IFN blockade model. Overall, scRNA-seq analysis offers valuable insights into the dynamic shifts in gene expression that govern tissue repair and regeneration following liver injury.

## Data Availability

The original contributions presented in the study are publicly available. This data can be found in the NCBI Sequence Read Archive (SRA) under BioProject accession number PRJNA1243372. The corresponding Biosample accession numbers are SAMN47619158, SAMN47619157, and SAMN47619156, with the related SRA accession numbers being SRR32904624, SRR32904626, and SRR32904625.
